# Evaluation of an Electronic Care and Rehabilitation Planning Tool With Stroke Survivors With Aphasia: Usability Study

**DOI:** 10.2196/43861

**Published:** 2023-04-17

**Authors:** Nadia Davoody, Aboozar Eghdam, Sabine Koch, Maria Hägglund

**Affiliations:** 1 Health Informatics Centre, Department of Learning, Informatics, Management and Ethics Karolinska Institutet Stockholm Sweden

**Keywords:** usability testing, stroke, aphasia, eHealth, rehabilitation, co-design, evaluation, user-centered design, effectiveness, user satisfaction, mobile phone

## Abstract

**Background:**

Patients with chronic illnesses with physical and cognitive disabilities, particularly stroke survivors with aphasia, are often not involved in design and evaluation processes. As a consequence, existing eHealth services often do not meet the needs of this group of patients, which has resulted in a digital divide.

**Objective:**

The aim of this study was to examine the effectiveness and user satisfaction of an electronic care and rehabilitation planning tool from the perspective of stroke survivors with aphasia. This would help us gain knowledge on how such a tool would need to be adapted for these patients for further development.

**Methods:**

Usability tests were conducted with 9 postdischarge stroke survivors with aphasia. Effectiveness was measured using task-based tests, and user satisfaction was studied through qualitative interviews at the end of each test. All tests were audio recorded, and each test lasted approximately 1 hour. The data were analyzed using qualitative content analysis. As the tool can be used by stroke survivors either independently or with some support from their next of kin or care professionals, the research group decided to divide the participants into 2 groups. Group 1 did not receive any support during the tests, and group 2 received some minor support from the moderator.

**Results:**

The results showed that the care and rehabilitation planning tool was not effective for stroke survivors with aphasia, as many participants in group 1 did not accomplish the tasks successfully. Despite several usability problems and challenges in using the tool because of patients’ disabilities, the participants were positive toward using the tool and found it useful for their care and rehabilitation journey.

**Conclusions:**

There is a need to involve patients with chronic illnesses more in the design and evaluation processes of health information systems and eHealth services. eHealth services and health information systems designed for this group of patients should be more adaptable and flexible to provide them with appropriate functionalities and features, meet their needs, and be useful and easy to use. In addition, the design and evaluation processes should be adapted, considering the challenges of this patient group.

## Introduction

### Background

Health information systems (HISs) and eHealth services have become essential parts of today’s health care. For a long time, the use of information and communications technology (ICT) in health care has been increasing. Sensors, electronic health records, home monitoring for older adults, wearables, and different assistive technologies have been designed for health care professionals, patients, and their next of kin. Telemedicine and the design of assistive tools for cancer care [1], cardiovascular diseases [2,3], and older adult care at home [4] are some examples in this area. Traditionally, patients and their next of kin have received eHealth services without being active in the development process. Nevertheless, their input in the design of the tools and services has become a crucial part of this context. The design techniques in health care have moved from traditional system design methods to interactive design methods with the users involved and focus on the interaction between them and eHealth services. In recent years, users have acted as partners in the development process, and co-design methods have become more popular in health care. Even though implementing this type of design in health care is challenging, the benefits are enormous. There is a growing interest in user involvement in the development of electronic health interventions, and patient participation has become an inseparable part of the design process [5]. Despite the fact that several attempts have been made to design HISs and services for patients [6], there is still a need for appropriate ones for different patient groups. Involving the end users is crucial in a user-centered design process [7,8] as it improves the understanding of the users’ needs and task requirements. It also plays an essential role in enhancing the iterations of the design and evaluation throughout the design process. Studies have shown that co-design improves patient knowledge, increases patient satisfaction, and improves care experience and sense of participation [9-12]. Several studies have focused on involving users and increasing their level of involvement in recent years [13-15]. Despite the growing popularity of involving patients with chronic illnesses with, for example, heart diseases, stroke, and obstructive pulmonary diseases in the design process [16-19], the number of eHealth services designed together with patients with either physical or cognitive disabilities or both is still low. This has resulted in a digital divide in which patients with the greatest needs do not have access to appropriate services [20,21]. Using a user-centered design and involving the end users could be a solution for reducing the digital divide for patients and citizens who are in need of accessing appropriate eHealth services to improve their care and rehabilitation processes.

Although ICT provides opportunities to improve, for example, the quality of health care [22,23], it also has its weaknesses and may affect health care and patients negatively [24]. Therefore, evaluation of HISs and eHealth services is recommended [24,25]. The formative evaluation of HISs and eHealth services is a crucial part of the user-centered design process. Recently, patients have been involved in several evaluation studies [26-30]. It is of great importance to design and evaluate HISs and services with and for patients to enable them, independently or with some support from their next of kin or health care professionals, to access and use the appropriate ICT tools that meet their needs. Studies involving older people with chronic diseases such as dementia have also shown the importance of the involvement of end users in the development of interventions as the usability results show an overall satisfaction with the platform [31].

The Medical Research Council guidelines for developing and evaluating complex health care interventions also stress the importance of formative evaluations and feasibility studies [32,33].

The clinical context of this study is stroke care. Stroke is one of the major global health problems causing death and adult long-term disabilities [34]. Stroke survivors often have several physical and cognitive disabilities and require care and rehabilitation from different care providers. Although they have long-term needs for support and rehabilitation and could potentially greatly benefit from the use of eHealth, this patient group is rarely involved in the design and evaluation of ICT tools. In addition, assistive technologies are often complex for patients with cognitive disabilities, and there are limited ones designed specifically for this group of patients [35]. A specific condition that many stroke survivors experience is aphasia, which results in difficulties in verbal expression, reading or writing, and understanding what others are saying [36,37]. Stroke survivors with aphasia are even less involved in the design and evaluation of different eHealth services. In the area of aphasia, there have been some studies focusing on the rehabilitation and speech-language treatment of patients with aphasia using ICT that show promising results [38,39]. In addition, a study focused on the key design features that can enhance the accessibility of mobile technology for people with aphasia [40].

In this study, we aimed to evaluate a care and rehabilitation planning tool, *My care plan*, that was designed and developed together with postdischarge stroke survivors (N=12) in our previous studies [41-43]. However, the patients participating in the design process were stroke survivors who did not have aphasia, and in this study, we aimed to explore if the tool could also be used by stroke survivors with aphasia or if it would need to be redesigned to meet their specific needs. Therefore, the participants in this study were patients who had a diagnosis of aphasia and were registered in a course for patients with aphasia in an education center in Stockholm, Sweden.

### My Care Plan

**Figure 1:**
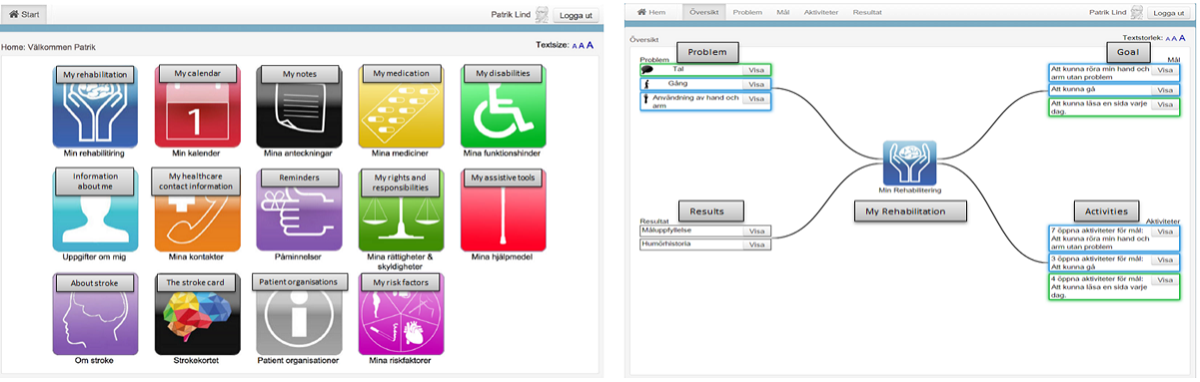
The home page and the overview page of the care and rehabilitation planning tool.

In previous studies [41-43], we designed an electronic care and rehabilitation planning tool. The development was conducted according to a user-centered design approach involving 12 postdischarge stroke survivors with milder physical and cognitive impairments. They were living at home and could handle computers. However, none of them had aphasia. The idea was that postdischarge stroke survivors, independently or with some support from their next of kin, should be able to use the tool throughout their care and rehabilitation processes at home. The tool consists of 2 main parts, namely, *My rehabilitation* and *Administrative and health related information*. *My rehabilitation* mainly focuses on establishing a rehabilitation plan by identifying problems and planning goals and activities (Figure 1). Currently, a neurology team consisting of a speech therapist, a counselor, a physiotherapist, and an occupational therapist visits a postdischarge patient of stroke and establishes a paper-based rehabilitation plan together with the patient and possibly their next of kin. The team, together with the patient, identifies the patient’s problems and defines and documents the intended goals and activities [44]. The idea with the electronic care and rehabilitation planning tool was that patients be able to access necessary information during their journey along with the establishment of a rehabilitation plan either independently or together with their next of kin or care professionals in a neurology team. Through the care and rehabilitation planning tool, the patients are able to document their problems and define their goals and related activities. As rehabilitation is a crucial part of the recovery process after a stroke, there is a need for a specific and clear goal-setting process. Therefore, we consciously decided to provide postdischarge patients with 2 different types of goals, namely, “simple” (eg, being able to talk to others) and Specific, Measurable, Achievable, Relevant, and Time-bound (SMART; eg, being able to read a book within 1 month) goals. Currently, the care professionals in neurology teams in Stockholm County work with almost the same types of goals and measure the patients’ progress using different scales depending on the patients’ problems and activities. However, in our care and rehabilitation planning tool, we used goal attainment scaling (GAS) [45] to quantify the achievement of the patients’ predefined goals. The expended goals, their importance, the difficulty level, the expected outcomes, and the baseline for the patient’s condition before the training are essential for using the GAS methods. Patients can independently or together with their next of kin or care professionals in a neurology team use a 5-point scale to obtain an overview of their achievements. The other part of the tool consists of administrative and health-related information, such as *my calendar, my notes, my medication, my disabilities, my care contacts, reminders, my rights and responsibilities,* and *my assistive tools*.

**Figure 2:**
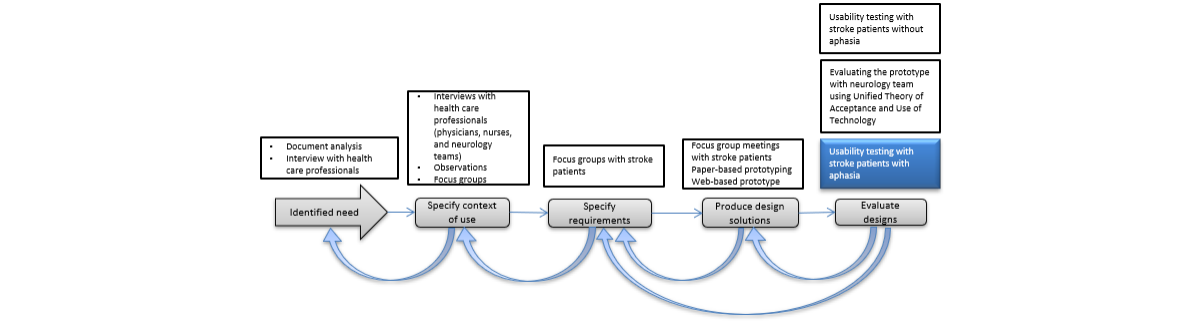
An overview of the design and evaluation process of the tool. This study is highlighted.

Postdischarge stroke survivors in the Stockholm County Council were involved throughout the requirement analysis and design process. The stroke survivors had milder physical and cognitive disabilities, were living at home, and could handle computers. The tool was designed using a user-centered design [46] approach and was developed based on the patients’ information needs throughout their care and rehabilitation processes. The design process started with interviews with health care professionals and focus groups with stroke survivors without aphasia. The patients’ information needs were collected, and appropriate eHealth services were identified. Paper-based prototypes were then designed together with postdischarge stroke survivors and discussed in further focus groups. On the basis of the feedback from participants, an electronic care and rehabilitation plan was then developed, and its preliminary version was evaluated with stroke survivors other than those who were involved in the design process. The electronic care and rehabilitation plan was then improved based on the feedback from the preliminary evaluation and additional focus groups with other postdischarge stroke survivors [43]. The final version was then evaluated from the care professionals’ perspective using the Unified Theory of Acceptance and Use of Technology [47]. Figure 2 illustrates the process of design and evaluation of the care and rehabilitation plan. In this study, we focused on evaluating the latest version of the prototype with a number of postdischarge stroke survivors with aphasia.

## Methods

### Overview

The electronic care and rehabilitation plan was evaluated through a number of usability tests. A usability test is a technique used in user-centered design for evaluating a product or service by testing it with representative end users [48]. The usability tests in this study were performed in April 2016 and will be described in the following sections, along with the participants and their recruitment.

### Description of the Usability Tests

A usability testing plan including 10 tasks focusing on finding information and establishing a rehabilitation plan by defining, for example, problems, goals, and activities was designed. In total, 3 pilot tests were performed with 1 usability expert, 1 patient of stroke, and 1 next of kin of a patient of stroke to validate the test and tasks.

Effectiveness and user satisfaction were the focus of this study according to the International Organization for Standardization 9241-11 guidance [49]. Effectiveness in this study focused on finding information and establishing a rehabilitation plan, including defining the problems, goals, and activities. Therefore, we explored the effectiveness of creating such a rehabilitation plan and finding necessary information in the care and rehabilitation planning tool. With regard to user satisfaction, we aimed to study user expectations, opinions, and preferences. We did not measure efficiency as participating patients had several cognitive and physical disabilities. In addition, the participants carried out the tasks while continuously thinking out loud throughout the whole task-based performance. This made the measurement of efficiency quite impossible. In total, 30 minutes were dedicated to task performance in each test. We used qualitative interviews instead of the System Usability Scale to measure user satisfaction as we were dealing with a group that had difficulties understanding what they read. Asking the participants to answer 10 questions with a 5-point scale rating would have been quite impossible as they had poststroke fatigue and several cognitive and physical disabilities.

Information letters were provided to all participants, and consent forms were obtained during the test sessions. A total of 4 tests were performed at Karolinska Institutet, and 5 tests were carried out in another location in Stockholm with participants who had difficulties getting to Karolinska Institutet. All sessions were audio recorded, and screen activities were video recorded using Camtasia Studio 8 software (version 8.4; TechSmith) for retrospective analysis by the research group. The computer used in this study was a Dell laptop running Windows 7 (Microsoft Corporation). The qualitative material obtained from the tests was transcribed verbatim and analyzed using an inductive content analysis approach according to Graneheim and Lundman [50]. All the authors were involved in creating the test tasks. All the tests were performed by the first author (ND). The first author (ND) went through the participants’ responses and conducted the preliminary data analysis. She grouped the responses into different categories. To ensure the credibility and trustworthiness of the results, the codes and categories were then discussed with other authors (AE, SK, and MH), and necessary changes were made. The process continued iteratively until a consensus on the categories and subcategories was reached. All authors were engaged in deciding the final codes and categories in this study. Prolonged engagement with the data, member checks, and the team’s unique combination of researchers with experience in qualitative research and evaluation studies allowed us to discuss and identify accurate codes and categories in this study. Quotations were extracted from the interview transcripts to illustrate core categories.

Each test took approximately 1 hour, and during each session, a short description of the study was given to the participants. Each test session was divided into 3 parts. The first part included an introduction to the test and receiving the consent form and the demographic questionnaire filled out by each participant. The consent form and introduction to the study and test were read by the moderator at the beginning of each test. The second part of the test included the tasks provided separately on paper and read aloud by the participants. During the last part of the test, participants were asked to answer some questions about different parts of the tool with a focus on user satisfaction and fill in a multiple-choice questionnaire based on resources from the work by Rubin and Chisnell [48] about the usefulness of the tool. The multiple-choice questionnaire consisted of 4 questions in which the participants, for example, expressed why it was necessary for them to have access to the care and rehabilitation planning tool and how useful it would be in their daily life. The test procedure for each participant is presented in Table 1.

**Table 1:** The procedure for the usability test for each participant.

Step	Activity	Description	Measurement	Instrument
Step 1	Consent form	Patients approved their participation in the study	N/A^a^	Paper form (read by the moderator)
Step 2	Questionnaire (demographic information)	Sex, age, time since stroke occurrence, experience of having an electronic or paper-based rehabilitation plan, experience of using ICT^b^, an overview of disabilities, and background (technical or nontechnical)	N/A	Paper form (filled with the help of the moderator)
Step 3	Introduction to the study and the test	Short description of the overall aim of the study and an introduction to the usability test	N/A	Paper form (read by the moderator)
Step 4	Introduction to the tool	Short description of the tool’s functionality	N/A	Video (short video of a screen recording with audio instructions)
Step 5	Test tasks	Part 1: finding information; part 2: establishing a rehabilitation plan	Performance effectiveness and completion of the tasks	Camtasia (TechSmith)
Step 6	Posttest interview	Questions on user expectations, opinions, and preferences	User satisfaction and open-ended questions	Paper form (read by the moderator)
Step 7	Posttest questionnaire	Questions about the usefulness of the tool	Multiple-choice questions	Paper form (read by the moderator and filled out by the participants)

^a^N/A: not applicable.

^b^ICT: information and communications technology.

### Study Participants

As the idea behind the design of the care and rehabilitation plan was that stroke survivors could use it independently or with some support from their next of kin or health care professionals, we decided to divide the participants into 2 groups (group 1 and group 2) to compare their performance in accomplishing the tasks. The participants were divided into the two groups based on the difficulties they faced and the level of support they received during the tests. Group 1 included patients who did not need any support and could follow the steps and accomplish some of the tasks successfully. In contrast, group 2 included patients that faced many challenges in performing the tasks and, therefore, received minor support during the test. In total, 6 usability tests of the *My care plan* tool were performed with 67% (6/9) of the postdischarge stroke survivors with aphasia, in which the patients did not receive any support from the moderator to perform the tasks (group 1). In addition, 3 usability tests were performed with the other 33% (3/9) of the postdischarge stroke survivors with aphasia (group 2). The patients in group 2 received some minor support from the moderator for performing the tasks. The support was mainly focused on asking questions such as “Do you see any rehabilitation plan?” or “Is that icon about assisting tool?”

The inclusion criterion for the participants in this study was that the time of stroke occurrence should not have been >5 years before. The participants had to live at their homes, have milder cognitive and physical disabilities, be able to talk with only minor difficulties, have aphasia, and be able to handle computers. Tables 2 and 3 provide an overview of the demographic information about the participants in this study and their experiences of, for example, having a rehabilitation plan or using different types of technology. The study was performed with patients with a diagnosis of aphasia from an education center in Stockholm.

**Table 2:** Participants’ characteristics (group 1), their experience of having rehabilitation and a rehabilitation plan and using technical devices, and a list of their disabilities.

Participant ID	Sex	Age range (years)	Time since stroke occurrence	Experience having an electronic or paper-based rehabilitation plan	Still receiving rehabilitation from neurology teams	Experience using technology	Current living situation	Cognitive or physical disabilities	Technical or nontechnical background
Participant A	Female	51-60	1-2 years	Paper-based rehabilitation plan	No	Computer and iPad	Partner	Speaking and writing difficulties and concentration problems	No
Participant B	Female	71-80	4.5 years	Paper-based rehabilitation plan	No	Computer	Alone	Speaking and writing difficulties	No
Participant C	Female	51-60	3 years	Paper-based rehabilitation plan	No	Computer and iPad	Partner	Right body side weakened	No
Participant D	Male	51-60	3 years	Paper-based rehabilitation plan	Yes	Computer, smartphone, and iPad	Partner, children, and other next of kin	Half-sided paralysis and speaking difficulties	No
Participant E	Male	61-70	2 years and 3 months	Paper-based rehabilitation plan	No	Computer, smartphone, and iPad	Partner	Aphasia	Yes
Participant F	Male	51-60	2 years and 9 months	No	No and had never received rehabilitation from a neurology team	Computers	Alone	Aphasia, writing problems, poor eyesight, and problem with right side of body	No

**Table 3:** Participants’ characteristics (group 2), their experience of having rehabilitation and a rehabilitation plan and using technical devices, and a list of their disabilities.

Participant ID	Sex	Age range (years)	Time since stroke occurrence	Experience having an electronic or paper-based rehabilitation plan	Still receiving rehabilitation from neurology teams	Experience using technology	Current living situation	Cognitive or physical disabilities	Technical or nontechnical background
Participant G	Male	41-50	2 years and 7 months	Oral rehabilitation plan	Yes	Smartphones	Alone	Half-sided paralysis	No
Participant H	Male	51-60	1-2 years	Paper-based rehabilitation plan	Yes	Computers	Partner	Speech difficulties, walking problems, and right arm and leg not fully functional	No
Participant I	Male	41-50	1-2 years	Paper-based rehabilitation plan	No	Computer	Alone	Writing and speech difficulties and balance and memory problems	No

### Overview of the Tasks

The effectiveness of the tool was studied for both groups of participants, namely, the 67% (6/9) of the participants who did not receive any support during the tests and the 33% (3/9) of the participants who received minor support for performing the tasks. A list of the tasks is shown in Textbox 1.

**An overview of the tasks in the study.**

- Task 1: find out which primary care center you are listed at.
- Task 2: find out if you have any problem in your rehabilitation plan.
- Task 3: add a new problem regarding your walking. Use the predefined International Classification of Functioning, Disability and Health (ICF) codes. Link it to occupational therapy.
- Task 4: add a new problem for your stress without using ICF codes. Link it to counseling.
- Task 5: add a simple personal goal. Add a description and link it to your stress problem.
- Task 6: add a new Specific, Measurable, Achievable, Relevant, and Time-bound (SMART) goal and link it to your walking problem. Choose *"as bad as it can be"* in step 3/4 and then choose “difficult” and “very important” in step 4/4.
- Task 7: decide on a new review point for your SMART goal for October 31. Choose *Klocka* 1 PM.
- Task 8: add a new activity for your SMART goal. The activity should start on November 27 *Klocka* 3 PM and be repeated every Thursday until December 18.
- Task 9: add your mood history for October 30, 2014, and November 15, 2014.
- Task 10: do the review for your SMART goal. Choose “Mycket bättre.”

### Ethics Approval and Consent to Participate

Ethics approval for the study was obtained from the Regional Ethics Committee of Stockholm on January 19, 2012 (2011/2093-31/5). Information letters were provided to all participants, and consent forms were obtained during the test sessions.

## Results

### Task Performance by Participants in Groups 1 and 2

The results of task performance by both groups are presented in the following sections.

#### Participants With No Support During the Tests (Group 1)

The analysis of the results from group 1 showed that only 17% (1/6) of the participants were able to complete half of the tasks successfully. The other 83% (5/6) of the participants had major difficulties performing the tasks, especially those related to the establishment of a rehabilitation plan. These tasks required practice, concentration, and several mouse clicks to be implemented. In total, 83% (5/6) of the participants were able to perform at least one of the tasks related to information seeking. A total of 17% (1/6) of the participants were not able to perform any of the 10 tasks. Table 4 provides an overview of task performance of the participants in group 1.

**Table 4:** An overview of the task performance of participants in group 1. The blank cells to show that some participants did not succeed in completing some of the tasks.

	Tasks									
	1	2	3	4	5	6	7	8	9	10
Participant A		✓		✓						
Participant B	✓	✓								
Participant C		✓					✓			
Participant D										
Participant E	✓	✓			✓	✓			✓	
Participant F		✓								

#### Participants With Some Minor Support During the Tests (Group 2)

Task performance was different for the second group of participants. All participants (3/3, 100%) performed the first 2 tasks on finding information without any support from the moderator. Generally, participants in both groups accomplished the information-seeking tasks more successfully than the rehabilitation tasks. This might be due to the large icons for different content, which made the necessary information visible and easy to access for the users. In group 2, most of the tasks related to the establishment of a rehabilitation plan (adding problems, goals, activities, mood history, and assessment of goal achievement) were accomplished successfully with some minor support and cues from the moderator (eg, *Is there any button you can push to add a new goal?*). Table 5 provides an overview of the task performance of the participants in group 2.

**Table 5:** An overview of the task performance of the participants in group 2.

	Tasks									
	1	2	3	4	5	6	7	8	9	10
Participant G	✓	✓	✓			✓	✓		✓	✓
Participant H	✓	✓	✓	✓	✓	✓		✓		
Participant I	✓	✓	✓	✓		✓	✓	✓		

The results of task performance in both patient groups showed that the tool is not effective for postdischarge stroke survivors with aphasia as most of the participants (6/9, 66%) were not able to accomplish most of the tasks. However, the results from group 2 show that all participants (3/3, 100%) were able to accomplish most of the tasks successfully with some minor support from the moderator.

### Qualitative Analysis of the Users’ Feedback

#### Overview

The content analysis of the posttest interview resulted in the 4 categories presented in following sections. The results in these sections are from participants in both groups. Textbox 2 provides an overview of the categories and subcategories identified in this study.

**An overview of the themes and categories.**

- Satisfaction
  Time to learn and support from next of kin
  The appropriate time for using the tool after stroke occurrence
- Design implications related to fatigue and concentration difficulties
  Information overload
  Complexity of concepts
- Visualization
  Using graphics
  Results visualization
  Using color-coding
- Perceived usefulness

#### Satisfaction

##### Overview

Despite the fact that the tool was not effective for postdischarge stroke survivors with aphasia, the posttest interview showed that the participants were positive toward using the electronic care and rehabilitation plan as a supporting tool for tracking their goals and activities. All participants except 1 (8/9, 89%) believed that they could or wanted to use the tool:

Yes, absolutely, I would like to [use the tool]. I use a computer every day, so I think it’s enjoyable [to use the tool]...It is fun to use the electronic care and rehabilitation plan. I think it is fun.

Participant A; group 1

Of course, it is nice [to use the tool]. There is a lot. There are things like this [information in the tool]. It is great.

Participant B; group 1

Sure, it was really difficult to understand but I do not know...I can use it.

Participant C; group 1

It would be great; it is easy to access things here.

Participant F; group 1

Some participants (3/9, 33%) believed that the tool was not difficult to use. However, most of the participants (6/9, 66%) found some parts of the tool quite complicated, for example, establishing a rehabilitation plan by adding goals and activities. A participant in group 1 believed that all parts of the tool were difficult, complex, and not easy to understand:

It was not so simple. It is a new program. You must learn it, it is pretty easy to click on [icons] and then go back and do it again.

Participant A; group 1

I think you could make it much easier for us [stroke survivors]. It was very difficult for me to choose between all things in each page...It was very difficult for me to choose between goals and activities. Very difficult to understand.

Participant C; group 1

##### Time to Learn and Support From Next of Kin

One of the participants mentioned that they could handle the tool if there was enough time to spend on working with the tool and, if necessary, they received some support from their next of kin:

I think I can [handle the tool]. If I work with it for a while, one day, so it will work. But as I told you sometimes my brother might help me.

Participant G; group 2

Although participants were positive toward using the tool, they believed that they needed time and training to learn and understand its different parts:

I think it [the tool] is very good. I need quite a long time. You need to sit and feel it. What is this so that you understand it?...You first should learn a little bit, then I think it is going to be pretty easy to understand and figure out what you want it to be like...I actually think that I am able to do it [use the tool]. Because now I do not have anything like this here, So It would be really great to be able to know how everything works.

Participant G; group 2

It takes time, I have noticed, but you have to dig to get the answer. You need to test it; it takes time for everyone.

Participant I; group 2

##### The Appropriate Time for Using the Tool After Stroke Occurrence

The patient journey model developed in our previous study [20] consists of different phases, such as “*At rehab clinic*” and “*At home*,” and events, such as “*Discharge from hospital*,” “*Discharge from rehab clinic,*” “*Coming home*,” and “*Clinical encounters*.” A phase may include several events in the patient journey. The participants in this study were in the “*At home*” phase. Some participants (2/9, 22%) believed that they would have benefited from the tool if they had access to it earlier in their care and rehabilitation journey as they had more severe communication difficulties in the beginning:

It would have been better [to have access to the tool] a bit earlier. I think when I was much worse when I could not talk at all, so it had been really great to look at the curve [the goal attainment scale curve]...In the beginning a lot was happening, three to four months, and then I noticed with my friends I improved a lot so I would benefit more [from the tool] then. I told my speech therapist that I want to have everything, so I am the kind of person that would love to have it [the tool].

Participant A; group 1

#### Design Implications for Patients With Brain Fatigue and Concentration Difficulties

##### Overview

Many patients experience brain fatigue and concentration difficulties after a stroke. Involving this group of patients in the design process and evaluating the care and rehabilitation plan together with patients who also had aphasia was challenging and required skills and knowledge about both design principles and physical and cognitive disabilities. On the basis of the data collected from the usability tests of the tool, different design implications were identified and are presented in the following sections.

##### Information Overload

As many stroke survivors with aphasia experience difficulties reading and understanding large amounts of text, designing any screen-based application such as our care and rehabilitation plan requires care. A home page with different icons contained administrative and health-related information. Some of the participants (3/9, 33%) were satisfied with the quantity of information on this page, but most of the participants (5/9, 55%) would like to have the possibility to choose only the information amount that met their needs:

It is easier to choose this [home page], but it is difficult to do other things. It [the home page] was great, it is very easy to use, e.g., rehabilitation, calendar and information about me, it is very good to find [this kind of information].

Participant C; group 1

It was pretty hard the whole thing in the beginning. It was a lot in this page [home page]...it is a little too much information (in home page). It would certainly be enough with five (icons). It is too much.

Participant F; group 1

It is good, you get help from these different things [information].

Participant E; group 1

Although the care and rehabilitation plan was designed based on the information needs of postdischarge stroke survivors, some participants in this study (2/9, 22%) did not find all information amounts in the home page necessary:

Indeed, reminder is necessary. Assistive tools, it is good, but I did not know what it is...You do not know what assistive tools means. I do not have any assistive tools so I do not need it, so I would not click it here.

Participant A; group 1

**Figure 3:**
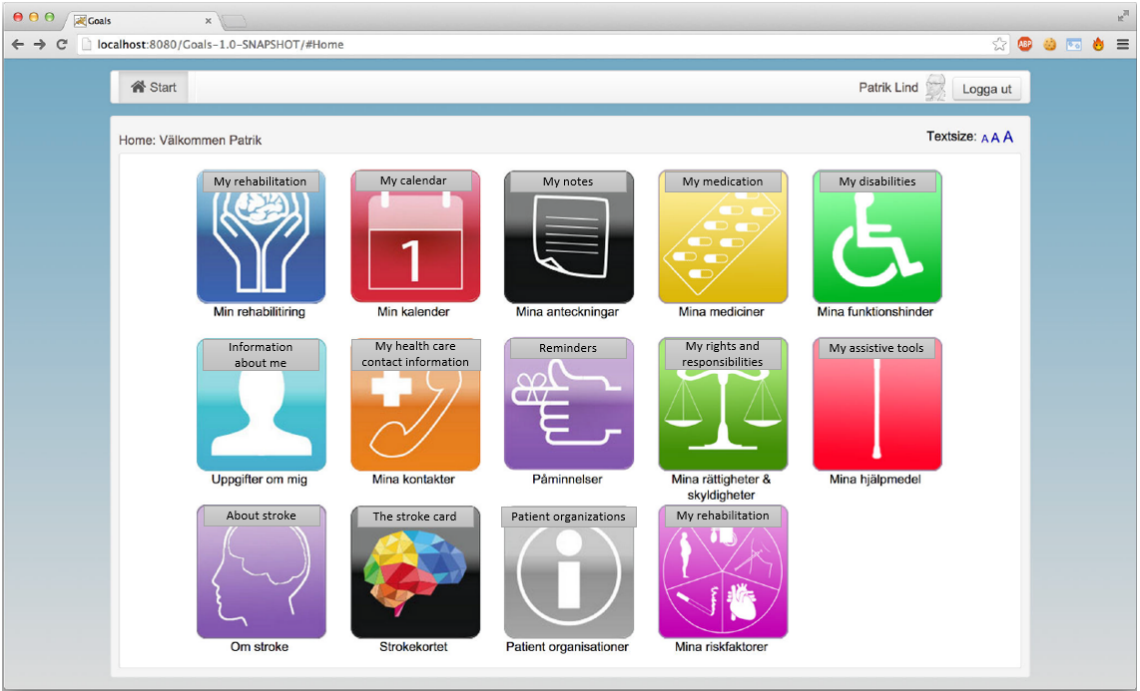
The home page includes different eHealth services (English translations of some necessary parts of the figure have been added).

The home page includes health and administrative information (Figure 3). Participants would like to be able to choose the necessary information based on their needs. They wished that the home page would include less information.

As some stroke survivors have bad eyesight, it was difficult to follow everything on different pages, for example, the overview page of the tool. However, big icons on the home page were perceived as easy to follow and access:

I actually think that it is good. Large buttons, so it is good. Even though I did not find one thing but like I said it was the first time [that I used the tool]. But it is good when they are so big [the buttons]. For some of us see a bit bad. We might see a bit like this on one side and this on the other side. So perhaps smaller icons...but this is great indeed.

Participant G; group 2

**Figure 4:**
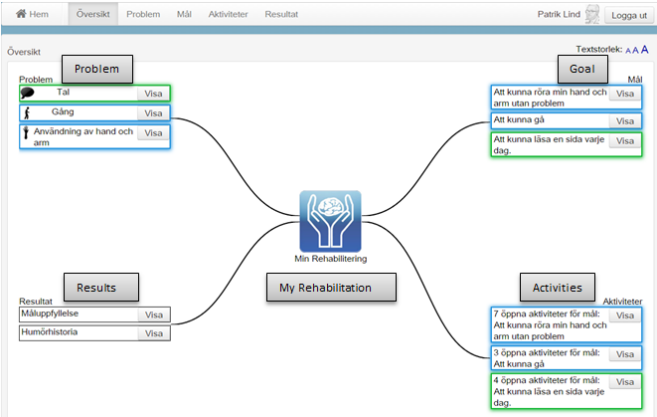
The overview page consists of an overview of, for example, problems, goals, and activities (English translations of some necessary parts of the figure have been added).

The amount of information on the overview page was also something that participants explained as difficult to understand and follow. The quantity of information on the overview page increases as the number of problems, goals, and activities increases (Figure 4). Some participants (3/9, 33%) were not satisfied with the increased amount of information on different pages, particularly on the overview page:

It is a bit like this: eee...wait, what is it now...? You wonder what all this is about and what is this...and I might have 6-7 goals and it gets very blurred.

Participant G; group 2

##### Complexity of Concepts

**Figure 5:**
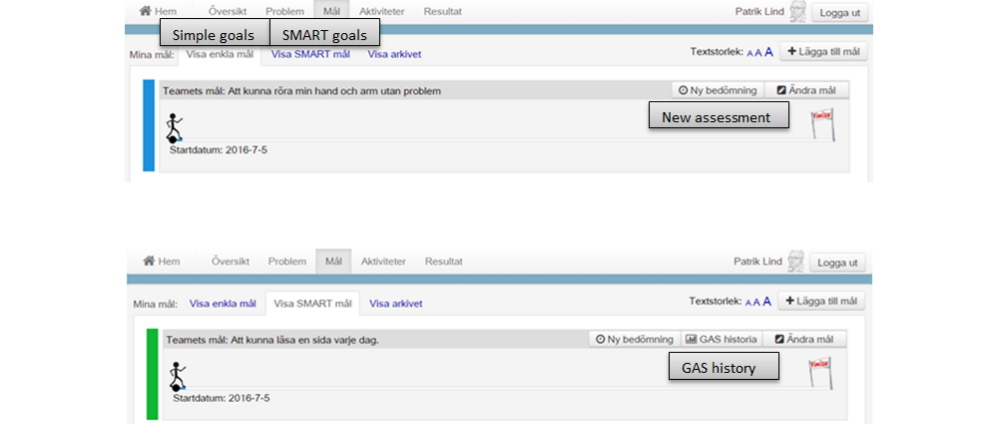
An overview of simple and Specific, Measurable, Achievable, Relevant, and Time-bound (SMART) goals (English translations of some necessary parts of the figure have been added). GAS: goal attainment scaling.

Almost none of the participants understood the difference between simple and SMART goals in the rehabilitation plan (Figure 5). Participants had different ideas about what a simple or SMART goal could mean. The difference in the description of the goals shows that the concepts used in the care and rehabilitation plan were not easy and clear to the participants. The participants wished to have some clarification on different terms and concepts on every page of the tool:

You can do simple goals e.g., at home, but I do not know what SMART goals mean.

Participant C; group 1

Results/goal achievement sounds really good. Mood history does not sound good.

Participant F; group 1

I do not know what a simple goal and a smart goal are...show simple goal?! What is this? Show smart goal?! You get so confused, and then my goals?!

Participant A; group 1

For smart goals you have a deadline.

Participant E; group 1

Simple and smart goals should be clarified, what is what...I do not understand [ICF codes].

Participant F; group 1

Simple goals you do without thinking about it, e.g., opening a door. You do it automatically. You do simple goals every day and smart goals are in future. It is about time.

Participant I; group 2

#### Visualization

Different parts, for example, goals and mood history of the electronic care and rehabilitation planning tool for postdischarge stroke survivors, were visualized using graphs, symbols, images, color-coding, and icons. The patients’ opinions on the visualization are presented in the following sections.

##### Using Graphics

Most participants (6/9, 66%) appreciated the graphs, symbols, and icons used in the care and rehabilitation planning tool. They believed that graphs and symbols supported them in understanding the process better:

I think it [mood history] is perfectly fine with such symbols, because you do not need to write a lot.

Participant A; group 1

The image (GAS curve) is good, you understand what happened immediately.

Participant F; group 1

Very good, big icons, different colors, text and images.

Participant I; group 2

Some participants (3/9, 33%) believed that the tool could include larger letters and figures. They had difficulties finding the necessary information for accomplishing some of the tasks:

[Headings] should be bigger e.g., activities, goals, problems. Large letters, so you can see them right away. I searched and did not see them, and I try the whole page and so on.

Participant A; group 1

I did not see this [the assessment point of the smart goal] perhaps you can see it if it was bigger. Particularly if it is bigger, now I cannot see it at all. I looked a lot on words [the menu at the top of the page], I searched a lot.

Participant I; group 2

##### Results Visualization

**Figure 6:**
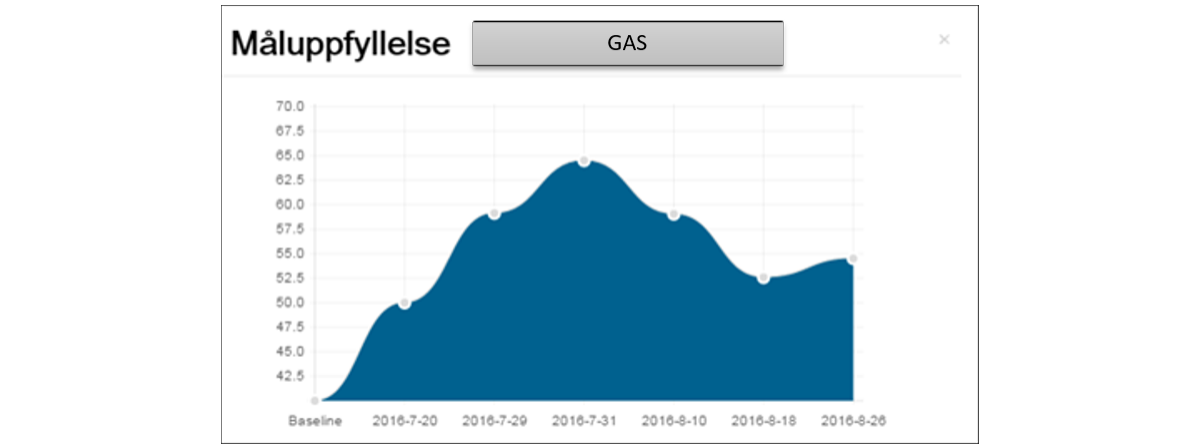
An example of a goal attainment scaling (GAS) curve (English translations of some necessary parts of the figure have been added).

Despite the fact that showing positive progress in goal achievement curves and mood history can motivate the patients to continue with their activities, negative results can also be a motivation factor that provides patients with an overview of their weaknesses. Goal achievements can be visualized in the tool as defining SMART goals and assessing them makes it possible for the user to obtain an overview in the form of a GAS curve (Figure 6):

Actually, it is good [negative results in GAS curve], for me it would be good to know what happens.

Participant F; group 1

It is great for sure, to understand how you have felt for a long time. It is better [to have it] so you can do something to make it a little better.

Participant B; group 1

It [mood history] is usually up there and if it looks like this [down curve] then I think what I have done, so I would think.

Participant A; group 1

Well, you have to dig, and then you seek after the truth when you know it. You know about your weaknesses, so this is not something new, you get it confirmed what you already know. You do something about the problem.

Participant I; group 2

Most participants (5/9, 55%) appreciated the mood history in the tool and were positive toward using the mood history in the care and rehabilitation planning tool. They believed that using this feature was easier than using other parts of the tool and liked the idea of having an overview of their mood history over time:

It is very good; it is much easier than the other parts and you understand how it works.

Participant C; group 1

**Figure 7:**
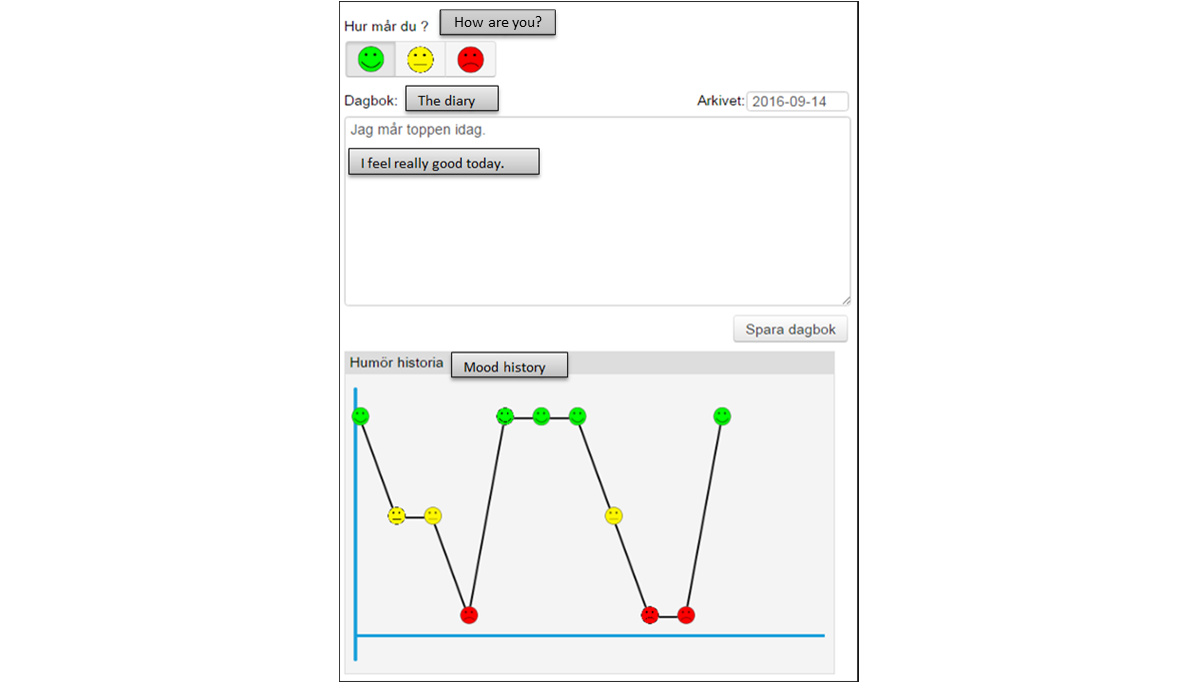
An overview of the mood history in the care and rehabilitation planning tool (English translations of some necessary parts of the figure have been added).

However, a participant believed that he would benefit more from a fatigue history than from a mood history (Figure 7):

For me it is fatigue. It does not happen so much with my mood, but it happens with fatigue.

Participant E; group 1

##### Using Color-Coding

**Figure 8:**
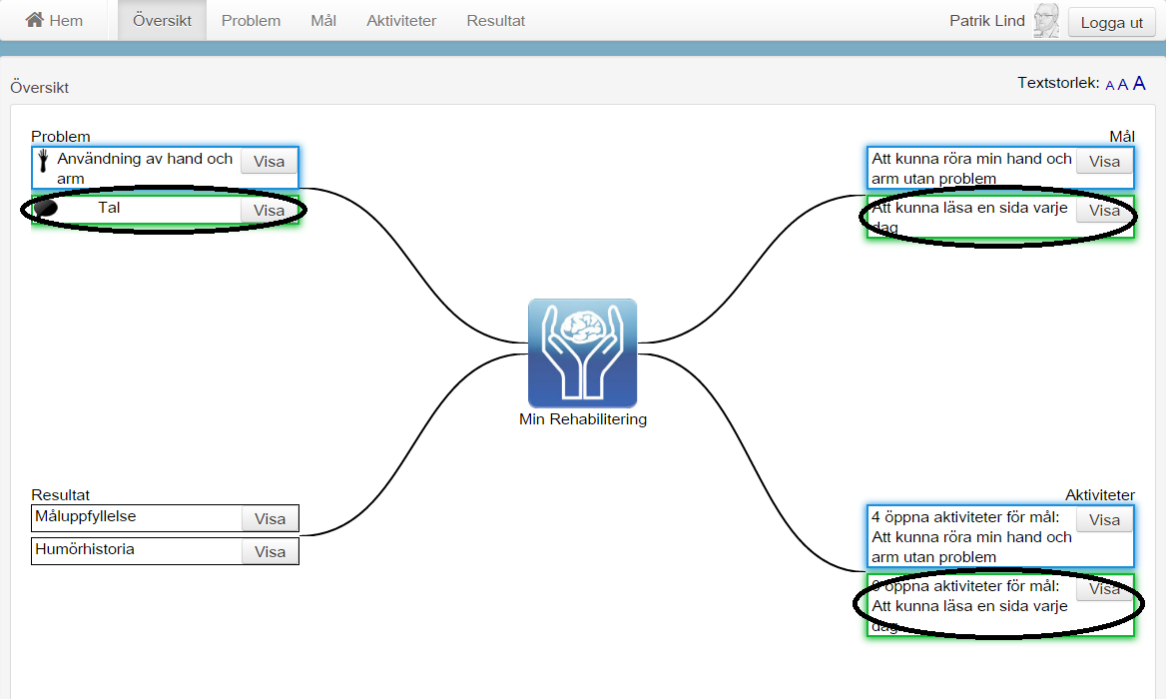
An overview of the connection between different problems, goals, and activities using color-coding.

In the tool, the goals, activities, and problems are linked in *My rehabilitation* using color-coding. Almost none of the participants understood the color-coding part of the tool before the description by the moderator. After clarification by the moderator, the participants were quite satisfied with the color-coding in the tool and believed that it helped them distinguish between different care professionals (Figure 8):

It is great, e.g., if I have occupational therapist then I have it in yellow.

Participant C; group 1

I think the color-coding is really good.

Participant F; group 1

Preferably bigger icon for rehabilitation, very large. It is quite small, there is a lot of space here, but it is good that it is same color and goals. Color-coding facilitates and helps a lot. Preferably bright colors, you can use different shades.

Participant I; group 2

#### Perceived Usefulness

Participants believed that having access to an electronic care and rehabilitation planning tool providing them with an overview of, for example, their problems, goals and activities, and administrative and health-related information would be useful for them throughout their care and rehabilitation processes:

I think it is good, because you can do it on computer, or it can be on TV and phone and because you know what you can do as well. Because I have different things, like for physiotherapy I go to Farsta (an area in south Stockholm) and then I have my speech therapist in Liljeholmen (an area near to central Stockholm) and with another care provider. Here, everything is gathered, and I think it is good, and then I have Alma courses and then you have everything here [in the tool]...Everything is gathered in one place.

Participant G; group 2

Participants believed that having access to the necessary information in the right place would motivate and support them in their planning throughout the care and rehabilitation processes:

It is good, it makes the life easier, a lot, and then above all, a very good overview, everything is there [information that patients need], e.g., my care contacts...and then my rights and responsibilities, it was interesting for me. You get information in the right place. It is fast. It is fast and easy in one place.

Participant I; group 2

[It is] available, motivational, at the same place, fun.

Participant A; group 1

It is nice [to have access to the tool], and it is all I need [the administrative and care related information].

Participant B; group 1

To be able to plan [having overview of goals and activities] and I can find everything I need [administrative and care related information].

Participant C; group 1

As many stroke survivors have memory loss, some participants (2/9, 22%) believed that using the tool would be helpful for remembering what activities they had done:

Yes, it can be good, and it might be better in retrospect when you can go back and see what you have done before e.g., that you have exercise walking for a while and for me it is to read etc. so you remember what you have done.

Participant E; group 1

## Discussion

### Principal Findings

Despite the fact that the participants were positive toward using the care and rehabilitation planning tool, the results showed that the tool was not effective for stroke survivors with aphasia. Participants in group 1 had challenges in using the tool and did not accomplish the tasks successfully. However, those in group 2 managed to accomplish most of the tasks receiving minor support from the moderator.

Participants mentioned that they needed time and training to understand the different parts of the tool. They even mentioned that they would have benefited from the tool if they had had access to it earlier as they had severe communication difficulties at the beginning of their care and rehabilitation processes.

As participants in this study had aphasia and difficulties reading and understanding large amounts of text, they wished to have less information on the home page and the possibility to choose only the information that met their needs. They liked the big icons on the home page but did not appreciate the increased amount of information on different pages of the tool. The use of concepts such as simple and SMART goals in the tool was not perceived positively, and the difference between them was not clear to the participants. Regarding visualization, most participants (6/9, 66%) appreciated the graphs, symbols, and icons used in the tool as they believed that they helped them understand the process better. The participants even believed that visualization of their mood history along with positive or negative progress in the goal achievement curves could be motivating factors for them to continue their activities and strengthen their weaknesses. However, color-coding used for connecting the goals, activities, and problems in *My rehabilitation* in the tool was not easily understood by the participants. Regarding the usefulness of the tool, the participants believed that having access to the tool would be useful to them throughout their care and rehabilitation processes as it provides necessary information that would motivate and support them during the entire journey.

### Comparison With Prior Work

Previous studies on the use of ICT for supporting patients with aphasia have focused on aphasia therapy, for example, helping patients with their fluency and voice disorders and identifying and determining management strategies for dysphagia [51,52]. However, the care and rehabilitation planning tool in this study focused on supporting this group of patients by providing them with an overview of their rehabilitation plan along with giving them access to necessary information that meets their needs throughout their care and rehabilitation journey. To our knowledge, there is no study focusing on the care and rehabilitation planning process for patients with aphasia and no study involving this group of patients in the evaluation process of an ICT tool. Martin et al [53] evaluated a software tool designed for older adults with aphasia and identified, for example, different design problems related to usability from speech and language therapists’ perspectives. In another study, Reeves et al [54] evaluated a multimedia application for patients with aphasia with 20 speech and language therapists. However, in our study, we evaluated the care and rehabilitation planning tool with patients with aphasia themselves and identified usability problems and the usefulness of the tool from the patients’ perspective.

In a previous study, care professionals’ perceived usefulness of the tool was examined using the Unified Theory of Acceptance and Use of Technology [47]. The results showed that, except for challenges such as time limitation and responsibility issues of the tool, care professionals were positive toward the tool and its potential usefulness throughout the care and rehabilitation processes of postdischarge stroke survivors. The results of this study also showed that despite some challenges, such as usability problems with the tool and patients’ several cognitive and physical disabilities, the patients were positive toward using the tool and appreciated its usefulness in tracking, for example, goals and activities, along with accessing necessary information. The researchers in this study were also aware of issues related to, for example, the responsibility of the system and technical support that need to be considered before the implementation of the system. However, the results of this study regarding participants’ satisfaction and their need to access necessary information and have an overview of their rehabilitation plan showed that designing and evaluating appropriate eHealth services is an increasing necessity in the care processes of patients with chronic illnesses.

Despite the fact that the tool was designed using a user-centered design approach with stroke survivors, there are still many design implications that need to be considered when designing for and with patients with chronic illnesses, particularly stroke survivors who have several physical and cognitive disabilities. The results of this study confirmed the general design implications, for example, information overload and visualization, for patients with cognitive disabilities discussed in previous studies [35,55]. The results showed that the tool designed for stroke survivors might not be effective for patients with aphasia after stroke. Therefore, involving this group of patients in the design process is of great importance even though it might be challenging for the research group and also for the patients. It is also of great importance to involve this group of patients in the evaluation process. Although involving this group of patients in this study was challenging and required skills regarding moderating the usability tests because of the patients’ speech and communication problems, it resulted in a better understanding of how to design appropriate eHealth services for this patient group.

One of the challenges in designing eHealth services for patients with disabilities, in this case, stroke survivors, is to consider their mental fatigue, neglect, and concentration difficulties. The amount of information on different parts of the tool was a problem during the tests. Most participants (6/9, 66%) became exhausted and frustrated using the tool. Some of them were distracted by the available information and started reading rather than performing the tasks in the test. Despite all these challenges and difficulties for participants and the research group during the tests, the participants were interested in using the tool as they believed that having access to necessary information gathered in one place and keeping track of their rehabilitation process are of great importance throughout their care and rehabilitation journey.

Although the results of this study showed that the tool is not effective for stroke survivors with aphasia as only 17% (1/6) of the participants in group 1, who in addition had a computer science background, was able to accomplish most of the tasks, we cannot draw any conclusions about the effectiveness of the tool based on this participant’s performance as their background might have affected the way the tasks were accomplished. However, providing some minor support resulted in greater effectiveness of the tool as most of the tasks were accomplished successfully by the participants in group 2. Overall, it was easier for most participants in both groups to perform the tasks related to finding information, but tasks related to the establishment of a rehabilitation plan consisting of several steps required more effort from the participants. However, for most patients, it was difficult to add their own goals and activities. Having support from participants’ next of kin was mentioned during the tests. Therefore, it might be easier for these patients to establish a rehabilitation plan together with their next of kin or health care professionals.

### Limitations

As only a few participants (3/9, 33%) were able to accomplish most of the tasks successfully, the care and rehabilitation planning tool should be more intuitive and adaptive, particularly when establishing the rehabilitation plan. Some participants in this study (3/9, 33%) mentioned that they would learn how to work with the tool if they had enough time and the opportunity to use it frequently. Therefore, it is of great importance to also evaluate the tool in the future to study its effectiveness after a certain period.

As this study was designed and conducted in 2016, the research group did not have access to the literature focusing on developing aphasia-friendly technology that has been published in recent years. To improve the system, in the next design or update round of the studies by Davoody et al [41], these studies [56-59] will be considered. These studies can be used to incorporate different elements of this research into the development or adaptation of the care and rehabilitation planning tool to assess its usability with people with aphasia.

### Conclusions

Although the care and rehabilitation planning tool is not effective for postdischarge stroke survivors with aphasia, it can be usable for this group of patients provided they receive some minor support from their next of kin or care professionals in a neurology team. However, eHealth services and HISs designed for these patients should be more adaptable and flexible to provide patients with appropriate functionalities and features and be useful and easy to use.

Despite the fact that involving patients with chronic illnesses with several physical and cognitive disabilities, particularly patients with aphasia, in the evaluation processes is a challenge because of their communication difficulties, their input for developing appropriate HISs and eHealth services is crucial. As the challenges existed throughout the user-centered design approach used for designing the care and rehabilitation planning tool, the design and evaluation processes should be adapted, considering these challenges.

Evaluating the care and rehabilitation planning tool from the perspective of this patient group provides insights into some of their information and communication needs. The results of this study show that patients with aphasia could benefit more from the tool if it could be adapted to their needs. However, further research is needed to confirm that the adjusted tool could be useful for this patient group.

In addition, to give different patient groups the opportunity to adopt the care and rehabilitation planning tool for their disabilities, different user profiles can be developed within the tool.

## Abbreviations

GAS: goal attainment scaling
HIS: health information system
ICT: information and communications technology
SMART: Specific, Measurable, Achievable, Relevant, and Time-bound
